# Influence of internal variability on population exposure to hydroclimatic changes

**DOI:** 10.1088/1748-9326/aa5efc

**Published:** 2017-03-28

**Authors:** Justin S Mankin, Daniel Viviroli, Mesfin M Mekonnen, Arjen Y Hoekstra, Radley M Horton, Jason E Smerdon, Noah S Diffenbaugh

**Affiliations:** 1 Lamont-Doherty Earth Observatory of Columbia University, New York, NY, United States of America; 2 NASA Goddard Institute for Space Studies, New York, NY, United States of America; 3 Emmett Interdisciplinary Program in Environment & Resources, Stanford University, Stanford, CA, United States of America; 4 Department of Geography, University of Zurich, Zurich, Switzerland; 5 Department of Water Engineering & Management, University of Twente, Enschede, The Netherlands; 6 Center for Climate Systems Research, Columbia University, New York, NY, United States of America; 7 Institute of Water Policy, Lee Kuan Yew School of Public Policy, National University of Singapore, 259770, Singapore; 8 Department of Earth System Science, Stanford University, Stanford, CA, United States of America; 9 Woods Institute for the Environment, Stanford University, Stanford, CA, United States of America; 10 Author to whom any correspondence should be addressed.

**Keywords:** climate change, internal climate variability, water resources, CESM large ensemble, blue water footprint

## Abstract

Future freshwater supply, human water demand, and people’s exposure to water stress are subject to multiple sources of uncertainty, including unknown future pathways of fossil fuel and water consumption, and ‘irreducible’ uncertainty arising from internal climate system variability. Such internal variability can conceal forced hydroclimatic changes on multi-decadal timescales and near-continental spatial-scales. Using three projections of population growth, a large ensemble from a single Earth system model, and assuming stationary per capita water consumption, we quantify the likelihoods of future population exposure to increased hydroclimatic deficits, which we define as the average duration and magnitude by which evapotranspiration exceeds precipitation in a basin. We calculate that by 2060, ~31%–35% of the global population will be exposed to >50% probability of hydroclimatic deficit increases that exceed existing hydrological storage, with up to 9% of people exposed to >90% probability. However, internal variability, which is an irreducible uncertainty in climate model predictions that is under-sampled in water resource projections, creates substantial uncertainty in predicted exposure: ~86%–91% of people will reside where irreducible uncertainty spans the potential for both increases and decreases in sub-annual water deficits. In one population scenario, changes in exposure to large hydroclimate deficits vary from −3% to +6% of global population, a range arising entirely from internal variability. The uncertainty in risk arising from irreducible uncertainty in the precise pattern of hydroclimatic change, which is typically conflated with other uncertainties in projections, is critical for climate risk management that seeks to optimize adaptations that are robust to the full set of potential real-world outcomes.

## 1. Introduction

Freshwater availability is a fundamental requirement for well-being. Because net availability is influenced by both supply and demand, factors like climate and geography, institutional norms and regulations, and urbanization and poverty all play important roles in determining regional and local water availability [1, 2]. Estimation of present and future water availability has received considerable attention [3–12]. Despite these important efforts, key uncertainties remain [7, 13–16].

Uncertainty in climate projections of anthropogenic warming from ensembles of climate models have three canonical sources: (1) scenario uncertainty, which arises from uncertainty about future greenhouse gas emissions trajectories; (2) model or structural uncertainty, which arises from different model choices and how those influence the climate response to forcing; and (3) internal climate system variability, which is the characteristic and natural inter-annual- to centennial-scale variations internal to the climate system [17, 18]. A number of studies attempt to sample such uncertainties by leveraging multi-scenario and multi-model ensembles like those from the Coupled Model Intercomparison Project (CMIP) [7–12, 15, 19]. As the principal source of climate projections used by the IPCC, CMIP is key to informing policy discussions on adaptation and mitigation, including assessment of future water resources that leverage CMIP output (e.g. refs. [8, 11, 12, 19]).

Despite important advances, partitioning the sources of uncertainty in multi-model ensembles of climate projections such as CMIP nevertheless remains a fundamental challenge because, to date, model uncertainty and representations of internal climate variability have been conflated within the multi-model ensemble [14, 20–22]. Furthermore, separating uncertainty associated with internal variability from model uncertainty is critical for real-world decision-making, as internal variability will ultimately determine the range of outcomes that could occur for a given forced response. Internal variability can thus confound expectations of anthropogenically-forced climate impacts (and therefore their optimal responses), potentially rendering adaptation decisions maladaptive over the short- and medium-terms.

While ‘robust adaptation decision-making’ seeks to guard against such possibilities, the strategy must optimize decisions against the range of possible real-world outcomes [23], such as those arising from internal variability, rather than those arising from model choices, which have no real-world analogue. Analyses of large single-model ensemble experiments reveal that simulated internal variability is sufficient to amplify, mask, or even reverse forced trends in atmospheric circulation, temperature, and precipitation over large spatial and temporal scales [14, 22]. The expression of internal variability in large single-model ensembles—a design heretofore unavailable in CMIP [20–22] and thus in many water resource projections forced by CMIP output—is sometimes called ‘irreducible uncertainty’, because the climate trajectories predicted by the different realizations are equally plausible, and independent of uncertainty arising from model structures or forcing pathways [14, 24]. It is therefore crucial to isolate robust estimates of internal climate variability in distributions of model-estimated future hydroclimatic impacts, which is currently infeasible with more computationally-intense hydrological and water resource models.

At the same time, however, hydrological and water resource models more credibly represent physical processes that influence the spatiotemporal patterns of water availability within basins and with greater fidelity than coarse Earth system models. The scientific and applied water resources community, including operational water supply forecasters and hydroelectric utilities, have long studied the historical behavior of modes of climate variability to understand and improve predictions of runoff variations [25–30]. Such work has the benefit of ensuring reservoir operations (and thus costs) are optimized given the climate state (e.g. [31]). It remains, however, that the influence of internal variability on water resources is largely characterized based on the short instrumental interval in a stationary climate. Internal climate variations that influence water availability occur on multidecadal and even centennial timescales, and such variability is poorly constrained by the instrumental record [32–34]. Furthermore, there are myriad sources of influential hydroclimatic variability that do not have well-characterized spatiotemporal structures and internal variability itself may be nonstationary in an anthropogenically-forced climate [35, 36]. The influence of such poorly-constrained multidecadal variability and the behavior of internal variability in a forced regime on water resources has received comparatively little attention and is the focus of this work.

**Figure 1.**
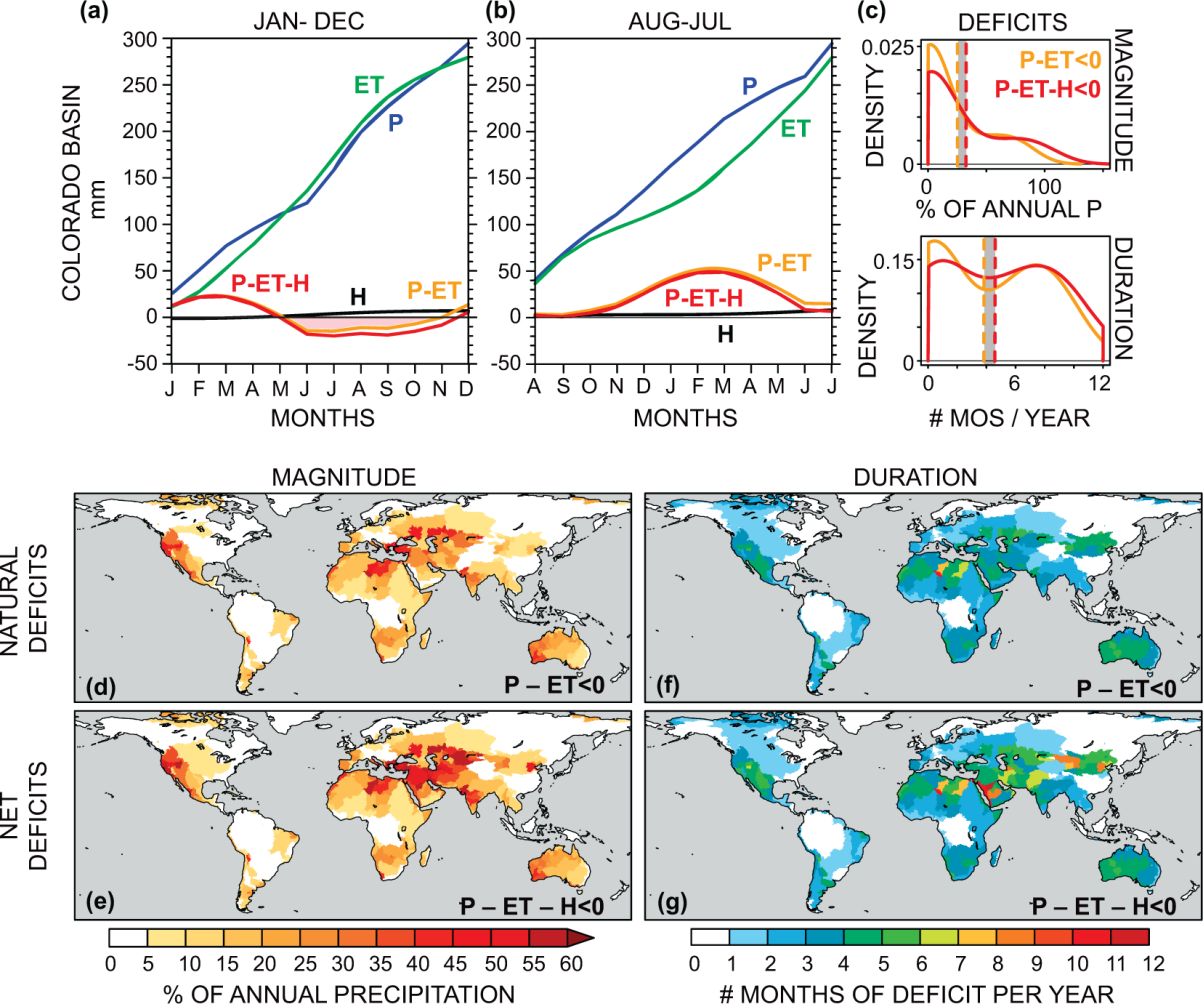
Observed natural and net hydroclimatic deficits. (*a*), (*b*), 12 month cumulative climatologies of precipitation (P), evapotranspiration (ET) from 1948–2010, and human blue water consumption (H), and their differences, P-ET and P-ET-H for the Colorado River basin, for the starting months of January [a] and August [b] in mm. The light red region in [a] shows Colorado’s P-ET deficit for the period beginning in January. There is no deficit in [b], highlighting that deficits are sensitive to starting month (methods). (*c*), PDFs of magnitude (top, as a % of annual P) and duration (bottom, # months per year) of Colorado’s deficits for all 12 starting months without (P-ET < 0, orange) and with (P-ET-H < 0, red) people’s consumption (H). The vertical dashed lines are the mean of the distributions and represent the ‘natural’ (orange) and ‘net’ (red) deficits plotted in (*d-g*). H shifts Colorado to greater mean deficits (grey bars). Average magnitude of natural deficits (P-ET < 0) (*d*) and net deficits (P-ET-H < 0) (*e*) (as a % of basin-scale total annual precipitation) calculated as the mean of each basin’s distribution of (*f*), (*g*), Average duration of natural (*f*) and net deficits (*g*) (as the number of months per any 12 month period).

Because our purpose here is to leverage a robust estimate of internal variability unavailable in hydrological or water resource models experiments, we use a framework that characterizes a basin’s mean hydroclimatic state using climatological precipitation and evapotranspiration from a single model in a single forcing pathway, *with* and *without* people’s water consumption—what we term *net* and *natural hydroclimatic deficits*, respectively (figure 1, methods). Additional structural and scenario uncertainties would emerge if we chose to use multiple climate models and radiative forcing scenarios, statistically- or dynamically-downscaled the climate model output [18], or used such output to force an offline water resources or hydrological model (e.g. refs. [7, 11, 12]). Finally, complex future water resource scenarios combined with such multi-model, multi-radiative forcing scenario output would articulate additional layers of uncertainties [8, 19, 37].

We seek to leverage the strengths and internal consistency of the Earth system model in representing climatological fluxes of precipitation and evapotranspiration, while avoiding additional uncertainties from downscaling or nested hydrological models and scenarios to represent basin-scale hydrology. Furthermore, we seek to quantify the uncertainty arising only from a robust estimate of internal variability and then contextualize that uncertainty within water resource scenario uncertainty arising from population growth trajectories. The perspective we take, therefore, is a long-term hydroclimatic one.

As such, we quantify the basin-scale uncertainty in hydroclimatic mean state changes arising from two sources: [1] irreducible uncertainty from Earth system model representations of multidecadal internal climate variability; and [2] three different population growth scenarios and their effect on water consumption. In doing so we explicitly account for how internal climate variability and population trajectories alone can influence the direction and magnitude of multi-decadal trends in future long-term hydroclimatic mean states within basins, and thus future population exposures to increased or decreased hydroclimatic deficits.

To quantify irreducible uncertainty from internal variability we use 30 members of the initial-condition ensemble (‘LENS’) of the NCAR Community Earth System Model (CESM) [22] run in the high RCP8.5 business-as-usual forcing pathway [38] (methods, figure S1 available at stacks.iop.org/ERL/12/044007/mmedia). The ensemble range comprises CESM’s estimate of irreducible uncertainty arising from persistent atmospheric circulation anomalies that propagate through the coupled climate system [22], such as jet stream placements. To quantify population scenario uncertainty, we combine a single scenario of per capita water use that is driven by three new Shared Socioeconomic Pathways (SSPs) of population growth [39]. Our analysis represents the first time these sources of information have been combined to constrain the influence of multi-decadal internal variability on population exposure to future hydroclimate deficits.

## 2. Data & methods

### 2.1. Natural and net hydroclimatic deficits

The basin-scale moisture balance includes the total ‘natural’ supply (P) and total demand (ET), as well as the water consumed by agricultural, domestic, and industrial uses (human-induced evapotranspiration or ‘blue water consumption’ (3), denoted H) (figures 1(a) and (b)). When cumulative water demand (ET or ET + H) exceeds supply (P), there is basin-scale net water loss (a deficit), and people and ecosystems draw water from stored sources (e.g. soil moisture, natural and managed reservoirs, snowpack, glaciers, groundwater, or inter-basin transfers). Changes in the duration and/or severity of these deficits could arise from changes in the environment and/or changes in human demand. Climatological precipitation minus evapotranspiration (P-ET) is thus a critical quantity determining the long-term surface moisture balance [40–42].

To quantify hydroclimatic mean states within basins, we calculate two metrics of natural and net hydroclimatic deficits to answer the following: how many months of any 12 month period does a basin have demand exceeding supply and what is the magnitude of that exceedance? In the present-day climate, such deficits encapsulate the managed and unmanaged strategies that ecosystems and people have to ensure water availability during times of deficit.

We define the hydroclimatic deficit *magnitude* and *duration* (or *E*[*D*]_*b*,magnitude_ or *E*[*D*]_*b*,duration_) as the average (expected) magnitude (in mm) and length (in months) by which cumulative demand exceeds cumulative supply in the basin (figures 1(c) and (d)). We calculate the hydroclimatic deficit magnitude and duration for each basin for two types of demand (figure 1), *without* human consumption (H) (called ‘natural’ deficits, P-ET) and with H (called ‘net’ deficits, P-ET-H) to highlight the human consumptive contribution to hydroclimatic deficit duration and magnitudes. In figures 1(a) and (b), the P-ET (natural deficit) and P-ET-H (net deficit) curves represent the difference between monthly-accumulating climatological supply (P) and monthly-accumulating climatological demand (ET or ET+H), for two different starting months, January and August. It is clear that the cumulative P-ET or P-ET-H curve can evolve differently based on the starting month (and thus the magnitude and duration of deficits), as illustrated for the Colorado basin in figures 1(a) and (b). We therefore average deficits across all starting months (January through December) to estimate a distribution of each basin’s magnitude and duration of deficit over any 12-month period by assuming basin water balance is 0 at the beginning of each 12-month interval (figures 1(c) and (d)). Formally, the hydroclimatic deficit magnitude, *E*[*D*]_*b*,magnitude_, is defined as:

$$E{\left[D\right]}_{b,\;\text{magnitude}\;}=\frac{1}{12}\sum_{k=1}^{12}\sum_{x_{i}}^{y_{i}}f{\left(D\right)}_{b,i,}$$

where *f*(*D*)_*b,i*_ is the natural (P-ET) or net (P-ET-H) curve for basin *b* in starting month *i*, where {i∣iℤ,1≤i≤12}, and *x*_*i*_ and *y*_*i*_ are the beginning and end of the deficit (where *f*(*D*)_*b,i*_ < 0 and > 0, respectively) for the curve. We then express the *E*[*D*]_*b*,magnitude_ as a percentage of basin-scale annual precipitation (%). We define the deficit duration similarly. It is the average number months for which the P-ET or P-ET-H curve is negative overall starting months *i*:

$$E{\left[D\right]}_{b,\;\text{duration}\;}=\frac{1}{12}\sum_{k=1}^{12}\sum_{i=1}^{12}\left[f{\left(D\right)}_{b,i}<0\right].$$

We show the *E*[*D*]_*b*,magnitude_ (figure 1(c)) and the *E*[*D*]_*b*,duration_ (figure 1(d)) for both natural (the vertical orange dotted line representing the mean of the P-ET<0 distribution) and net deficits (the vertical red dotted line representing the mean of the P-ET-H<0 distribution) for the Colorado basin. The distance between the orange and red dotted lines in figure 1(c) and (d) illustrates the increased deficit magnitude and duration from human water consumption in the Colorado basin. Such an accounting provides a stable climatological method to annualize monthly-scale cumulative P-ET and control for hydrological heterogeneities across basins, such as differential water years and seasonal cycles.

We note that the deficits do not need to be in consecutive months—the calculation is simply the average magnitude and duration by which total demand exceeds total supply in any 12 month period. We define ‘severe’ deficits as those that exceed either 50% of annual basin scale precipitation in magnitude (i.e. more than half of annual basin supply) or 6 months of the year in duration (i.e., more than half the year in deficit). Note that because this deficit metric is a climatological characterization of basin supply and demand, even modest changes in its magnitude or duration would likely necessitate management responses.

This measure of hydroclimatic deficits is an approximate index of runoff availability. It must be noted however that our measure assumes negligible changes in storage, a simplification that would bias water resource estimates for basins with large grid cell fractions of lake cover, aquifer systems, or ice fields. Furthermore, P-ET does not allow us to assess potentially critical changes sub-seasonal watershed hydrology. Nevertheless, P-ET is a meaningful first-order measure of hydroclimatic mean states that is routinely used in large-scale global climate model-based studies of water resources [41, 42].

### 2.2. Observational and reanalysis data

Hydrological basin demarcations come from a modified version of the Simulated Topological Network (STN-30p), which represents the spatial extent of river drainage basins globally at 0.5° resolution [43] (see supplemental). We aggregate all gridded data to the area-weighted basin scale.

We use version 2.0 of the Global Land Data Assimilation System (GLDAS-2) as our baseline climatology (1948–2010) of P (the sum of variables Rainfsfc and Snowfsfc) and ET (variable Evapsfc). GLDAS-2 uses meteorological assimilation and hydrological simulation to create a 0.25° reanalysis of observed land-surface processes for 1948–2010 [44] that closes the annual basin-scale water budget (figure S1(d)). For additional human-induced evapotranspiration (H), we rely on the blue water footprint (BWFP), a gridded, 5-arc-minute-resolution (~10 × 10 km^2^ at the equator), monthly climatology of people’s consumptive demand of surface and subsurface water estimated from 1996–2005 [3].

This method avoids the double counting of ET in adding BWFP to naturally-occurring ET estimated from GLDAS. GLDAS uses the NOAH land surface model, which employs the Penman-Monteith equation to estimate actual ET. It does not take into account human irrigation, which is the principal source of human water demand globally. NOAH uses the land cover classification with a single crop type and a single evapotranspiration parameter [45]. Therefore, GLDAS only includes the productive human-use of naturally occurring ET (or what is termed the green water footprint), such as for rain-fed agriculture. The GLDAS and BWFP thus represent distinct sources of ET.

### 2.3. Climate model data

We analyze projections of P and ET from the Community Earth System Model Large Ensemble project (CESM LENS) [22], which simulates the coupled atmosphere-ocean-land-sea-ice system from 1920–2080 at a 1° resolution (displaced pole grid, gx1v6), using the RCP8.5 forcing pathway [46]. The experimental design uses a single model to simulate climate 30 times in a single forcing pathway, with minor perturbations in atmospheric conditions prescribed at initialization in the year 1920. These runs are initialized from identical ocean, land and sea-ice states drawn from asingle CMIP5-type1850–2100 simulation (which itself is initialized from a post-equilibrium 1500-year preindustrial control simulation). The range of climate states represented in the ensemble provides CESM’s estimate of irreducible uncertainty induced by uncertainty in the atmospheric initial conditions and how that influences persistent circulation patterns, and thus mean hydroclimate [14, 21].

We use prognostic P and ET to calculate natural and net deficits for each basin in each of the 30 CESM LENS realizations, which explicitly accounts for changes in P or ET, and thus land water balance, arising from simulated precipitation phase changes, snowmelt timing, and other complicating surface hydrology features. For P, we use the sum of convective and large-scale precipitation (PRECC + PRECL, m s^−1^), and for ET we use the sum of terrestrial and canopy evaporation, and vegetative transpiration (QSOIL + QVEGE + QVEGT, m s^−1^). For each realization, we calculate linear trends estimated on monthly and annual area-weighted basin-scale output from 2011–2080 Using the full 2011–2080 series (70 yr of data), we fit autocorrelation-corrected generalized least squares (GLS) trends to each month and each ensemble member based on the autocorrelation structure of the residuals, following ref. [21]. We express each month’s basin-scale trends as a relative change (% per 50 yr) from each ensemble member’s 1948–2010 baseline mean. Following ref. [47], we multiply each basin’s CESM trends by the monthly GLDAS baseline mean to get 30 estimates of absolute change relative to the GLDAS baseline mean and add these absolute changes to the GLDAS mean, providing 30 estimates of climatological monthly P and ET by 2060. Signal-to-noise ratio is calculated by comparing the magnitude of the mean of 30 individual trends (signal) to the standard deviation of those trends (noise), with the ensemble signal considered to be robust if the signal-to-noise (S/N) ratio exceeds one [21].

### 2.4. Population data and projections

**Figure 2.**
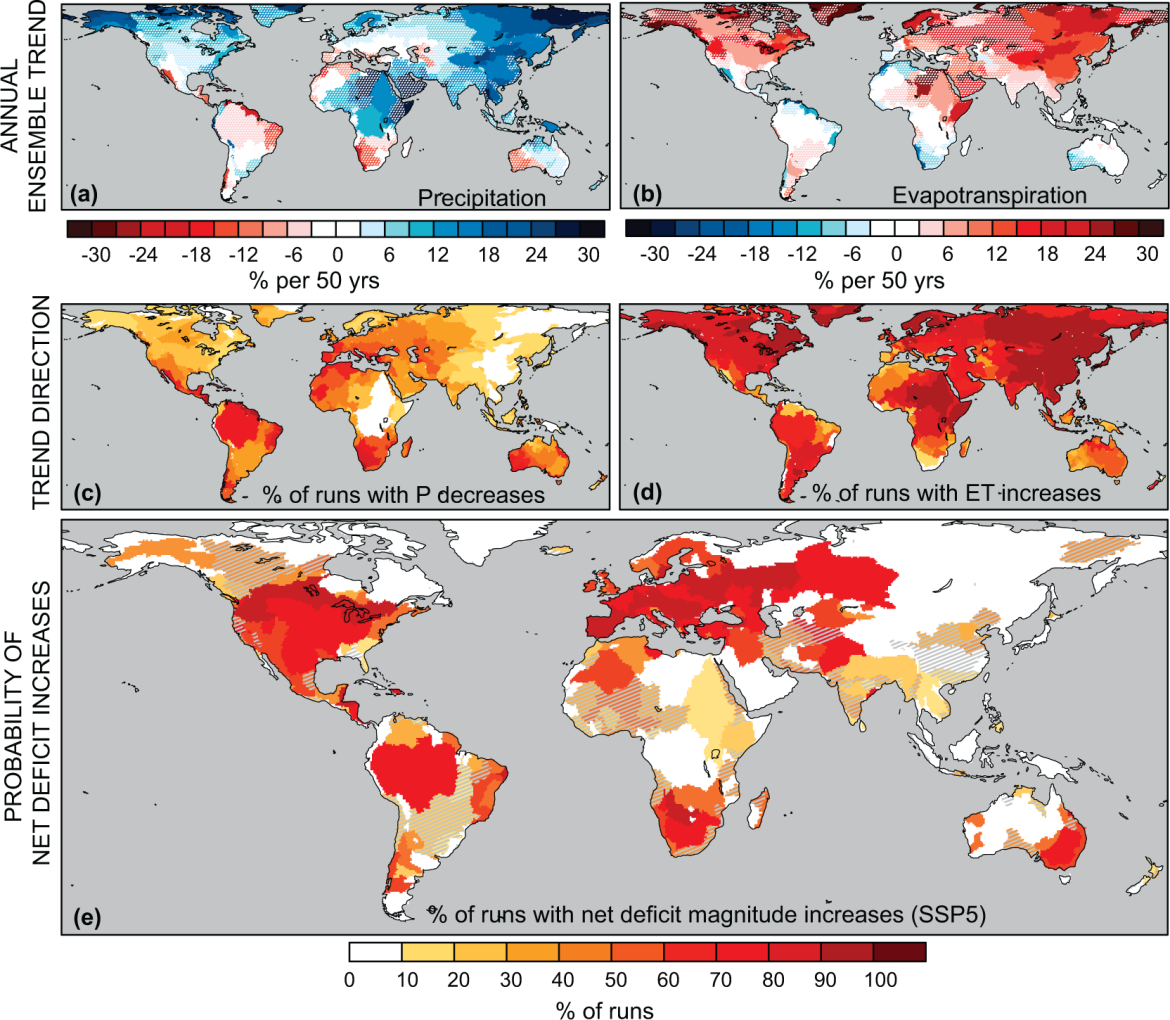
P and ET trends and the probability of net deficit increases at 2060. (*a*), (*b*), Linear basin-scale trends in mean annual P [a] and mean annual ET [b] estimated from 2011–2080 Solid colors in [a] and [b] indicate basins where the ensemble signal-to-noise (S/ N) >1; stippled basins indicate where the ensemble S/N <1. (*c*), A measure of ensemble variability in the sign of trends, indicating the percent of ensemble members with linear P trends <0. (*d*), As in [c], but the percent of ensemble members with linear ET trends >0. (*e*), Percent of the 30 ensemble members exhibiting an increase in the magnitude of net deficits at 2060. We apply a threshold for inclusion for each run in each basin: the absolute magnitude of net deficit change must be greater than present-day water storage infrastructure in the GRanD database (methods). Solid basins in [e] show significant ensemble mean change (*p* < 0.05) while hatched basins indicate ensemble mean changes that are insignificant based on a one-sample Student’s t-test. See supplemental for the probability of duration increases and other SSPs.

We use present-day gridded population estimates (2015) from the Center for International Earth Science Information Network [48]. To project future BWFP from population changes, we use the global spatially-explicit future population estimates from ref. [39] that presents decadal average populations based on the Shared Socioeconomic Pathways (SSPs) narratives. We use the three population projections (SSP2, SSP3, and SSP5, respectively) that could be consistent with RCP8.5 [49], the emissions pathway with which the CESM LENS was forced. Using present-day BWFP and populations, we estimate basin-scale per capita water demand and project three estimates of basin-scale monthly water demand using the future population estimates for each SSP, assuming stationary per capita consumption (supplemental). We then calculate deficits as outlined above. We also examine the sensitivity of these projections by calculating the range of future net deficits assuming no change in consumption or population. For figures 2(e), and S3 and tables S1 and S2, we include a threshold for net deficit increases that must exceed present-day artificial storage reservoir capacity within the basin from the Global Reservoir and Dam (GRanD) Database [50]. We aggregate the variable CAP_MCM to the basin scale and normalize that value to the basin area.

We provide statistical tests of significant change at the global and basin scale. In table 1 we present the global tests of significant change for all variables across all scenarios. We use three non-parametric tests of significant change using an a-level of 0.05 (Pr(>|*ϕ*|) < *α*, where *ϕ* is the test statistic: (1) the Welch’s t-test, (2) the Mann-Whitney, and (3) the bootstrapped Kolmogorov–Smirnov (KS) test. At the basin scale, statistical significance is calculated using the standard one-sample Student’s *t*-score given a preselected *α*-level: Pr(>|t|) < *α*, using *α* = 0.05. ‘Probability’ quantification is the percent of the ensemble agreeing on a consistent direction of change above a threshold of magnitude [51, 52].

## 3. Present-day natural and net deficits

Based on a 1948–2010 climatology from the GLDAS-2 surface reanalysis, 415 of the 676 basins we examine show at least one month of natural deficit on average, and 278 basins have natural deficit magnitudes equal to at least 10% of their annual precipitation (figures 1 (d) and (f)). On average, the moist tropics and low-ET high-latitudes spend little to none of the year in natural deficit, compared with nearly a quarter of the year for monsoon regions, and nearly half the year (with magnitudes >25%) for global drylands.

The seasonal interactions among P, ET, and H fundamentally change the basin-scale supply and demand characteristics (table 1, figures 1(c)–(g) and S2). At the global scale the impact of human present-day water consumption, H, is to double the observed basin-mean natural deficit magnitude of ~11%, while increasing the global-mean duration by 15%, both statistically-significant changes (table 1, figure S2). For many basins, the effect of human water consumption is even more pronounced: present-day human consumption within the Colorado basin increases naturally-occurring deficits by ~30% in magnitude and three-quarters of a month in duration (the grey bars in figure 1(c) and (d)). In the Indus basin, deficit duration increases by ~4.5 times and deficit magnitude by ~11 times (figures 1(f) and (g)). Likewise, irrigation demand on the North China Plain (along the sub-basins of the Yellow River) doubles both the magnitude and duration of natural deficits (figures 1 (d)–(g)). In the agriculturally-intensive San Joaquin basin in western North America, human water consumption more than doubles deficit magnitudes, intensifying an already arid regime (figures 1(d) and (e)).

## 4. Future natural and net deficits

Using LENS, we project basin-scale changes in monthly and annual P and ET over the coming decades (figures 2 (a)–(d) and S1). These natural deficit projections are coupled with estimates of population-based H to calculate net deficit magnitudes and durations for three SSP future population scenarios (figures 2(e) and S3, methods). We calculate the ‘probability’ as the percent of the ensemble showing a consistent direction and minimum threshold of change [51, 52]. The threshold we employ here is a conservative one: net deficit magnitudes increases that exceed present-day artificial reservoir storage capacity within the basin, based on the GRanD database [50]. At the global scale, the increased magnitude and duration of net deficits is driven by increases in both basin-scale mean evapotranspiration and human water demand across all three SSPs (table 1).

The risks of increased net deficits are large (figure 2, S3, tables S1, S2). Examining the likelihoods of increases in net deficit magnitude that exceed present-day reservoir storage capacity, we find that approximately a quarter of basins globally (~31%–35% of future population) have >50% probability of increased net deficit magnitudes, while 17% of people will be exposed to >75% probability, and up to 10% of people will be exposed to >90% probability (figures 2 (e) and S3, table S1). The possibility for decreased net deficits across the ensemble is also large: populous basins in East Asia and Central Africa, for example, show likely (>66% [52]) decreases in net deficit magnitudes (figures 2(e), S3), attributable to increases in P that exceed increases in ET (figure 2(a)). Furthermore, up to 13% of people are projected to live in the 10% of basins in which the probability of increases in net deficits is nearly equal to the probability of decreases.

**Figure 3.**
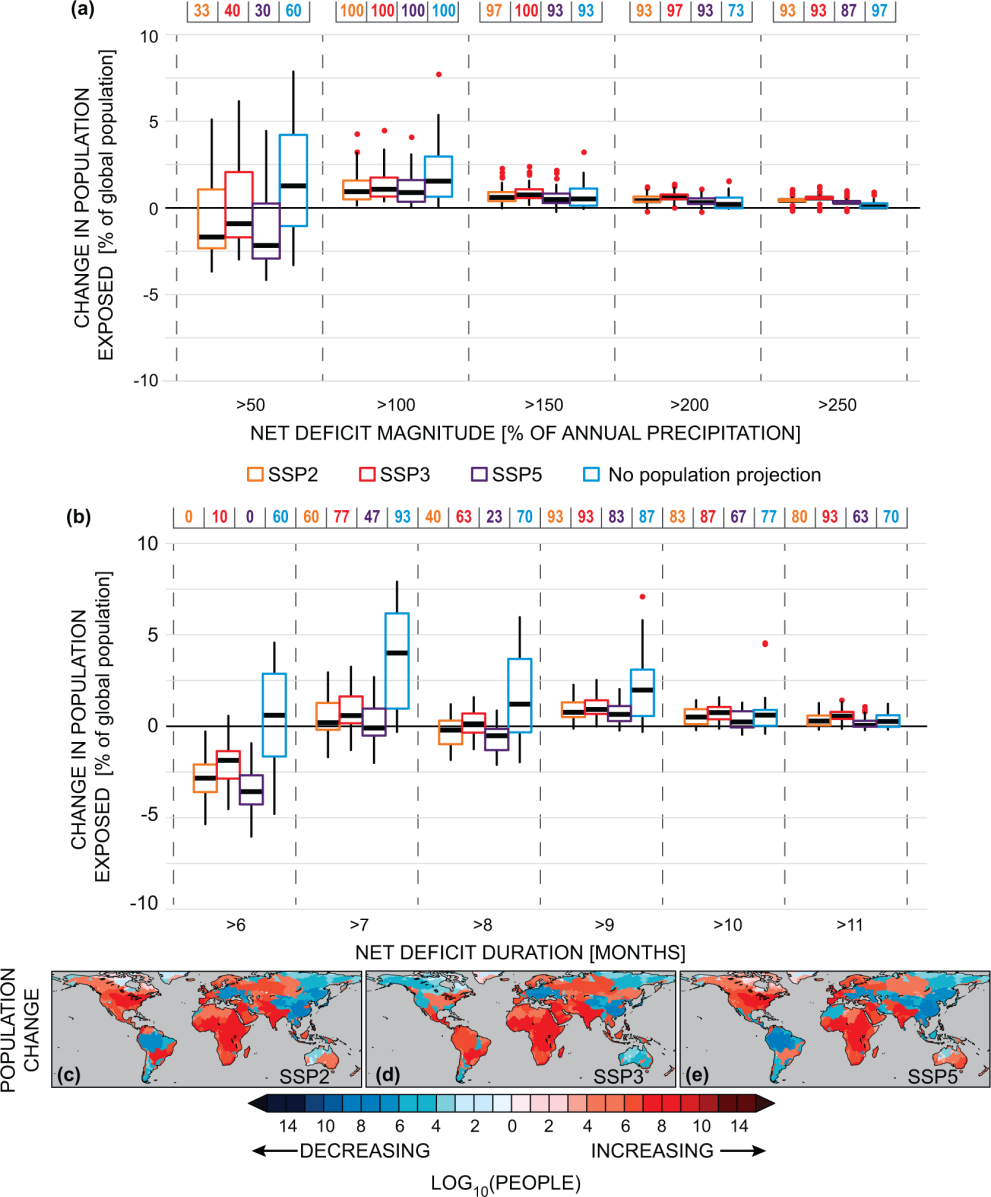
(*a*), (*b*), Irreducible uncertainty in changes of population exposure to net deficits of differing magnitudes [a] and durations [b] by 2060. For 3 SSPs congruent with RCP8.5, (SSP2, orange, SSP3, red, SSP5, purple) we show the ensemble range in the change in the number of people (in % of global population in each scenario) exposed to more than the net deficit value in each bin (>X) as a box plot (25th, 50th, and 75th percentiles, with outlying ensemble members shown as red dots). The blue box plots in [a] and [b] shows the effect of climate change only on population exposure (i.e. the change in population exposure if there were no population human consumption changes). Colored numbers across the top of each panel indicates the percent of the ensemble projecting increased population exposure for each scenario in that bin. (*c*)–(*e*), Basin-total population changes (logged) by 2060 for SSP2 [c], SSP3 [d], SSP5 [e] from the CIESIN 2015 estimates. Blue values indicate decreases, red, increases. Note the irregular spacing of the color bar for values >10 and spatial variability in population gains and losses across SSPs.

To examine the implications of irreducible climate uncertainty on future population exposure to hydroclimate deficits, we calculate the distribution of population exposed to different levels of severe net deficits by 2060 (figures 3 (a) and (b)). Consider the exposure range for net deficit magnitudes of >50% (first bin, figure 3(a)): if there were no population change (and no attendant demand changes), the ensemble predicts 60% probability that more people will be exposed, but the irreducible range of global population exposure is −3% to 7%. Across the SSPs, the ensemble projects a median decrease of 1.5% of global population exposed, with up to an additional 6% of people exposed, as in SSP3 (figure 3(a), first bin). Further, depending on the individual SSP, the range of irreducible uncertainty in exposure can span up to ~9% of global population as in SSP3 (figure 3 (a)). As progressively more severe deficits are considered, however, the irreducible uncertainty diminishes, and the ensemble predicts additional people exposed to net deficit magnitudes and durations relative to today (figures 3(a) and (b)).

## 5. Discussion & conclusions

Within each SSP, the range in predicted population exposure is entirely an expression of persistent multidecadal variability in P and ET (figures S4, S5). Complicating the picture of ‘robust change’ in hydroclimate is the potential for P and ET to have statistically-significant basin-scale trends in either direction, far from the ensemble mean response (figure S5). Moreover, variability in LENS hydroclimatic trends can span a majority of the inter-model variability in CMIP5 trends, particularly for populous mid-latitude basins [47], suggesting that some of what is considered model uncertainty is actually internal variability. A robust evaluation of this hypothesis requires larger ensemble simulations with each CMIP model, which is currently unavailable. Thus the LENS provides the most complete estimate of hydroclimatic variability in a transient simulation available, with its distribution representing the most meaningful analog to date for the potential range of real-world uncertainty around any climate and/or water-use scenario.

In contrast to the uncertainty within SSPs, which is climate-driven, the uncertainty across SSPs is a function of where future populations will aggregate or diminish (figures 3(c)–(e)). While the uncertainty across the SSPs in predicted exposure can be sizable in absolute terms (particularly in comparison to the scenario of no population change, which is largely driven by population decreases in East Asia across the SSPs (figures 3(c)–(e)), there is considerably more agreement across SSP projections than within them (figures 3(a) and (b)).

The uncertainty in future net deficits thus appears more driven by uncertainty in future hydroclimatic change than the uncertainty in future population-only driven demand (table 1 and figures 2, S2). This conclusion is emphasized by two results: First, there is the large directional uncertainty in natural deficits, which by definition, neglect human demand: 78% of basins (presently home to 83% of people) exhibit both increases and decreases in magnitude across the ensemble, while 76% (presently home to ~80% of people) exhibit both increases and decreases in duration (figure S4). Second, across the three population scenarios, the relative population exposed to increased net deficit magnitudes or durations shows little variation, consistent with other studies of population exposure to climate extremes [53] (figures 3(a) and (b) and tables S1, S2). Uncertainty in future human water demand from population changes *alone* is therefore largely insufficient to generate directional uncertainty in future net deficits, though it may amplify or diminish uncertainty arising from climate variability. Changes in per capita water consumption profiles, which we explicitly do not examine here, would likely reduce the number of basin subject to irreducible directional uncertainty in future net deficits. Identification of this would require a large ensemble of water resource model simulations with multiple scenarios of future human water consumption.

It is important to note that we do not explicitly quantify whether populations sustainably manage their deficits, nor the social responses that could offset future deficits. Furthermore, because our estimates are made at basin scales and represent hydroclimatic mean states based on long climatologies, such mean changes potentially belie water stresses at finer spatial and temporal scales, such as changes in seasonality, extremes, and the like. However, because our measures of net hydroclimatic deficits characterize long-term water availability, the risks of change we identify in them underscore the importance adaptive and flexible water management and storage for future human water demand. Depending on the characteristics of the basin, storage can come from soil moisture, groundwater aquifers, snowpack, glaciers, built infrastructure or inter-basin transfers. These managed and unmanaged systems play a critical role in fulfilling environmental and human requirements during months of hydroclimatic deficit [54]. Our results suggest that, for many places, these systems will need to be adjusted in order to fulfill water demands in the future. These adjustments are likely to require reconciliation between the social, political, cultural, institutional, and infrastructural realities of each basin. Our results also highlight the need for a greater representation of multidecadal internal climate variability in finer-scale water resource projections, which is critical to ensure water managers are prepared for the broadest set of possible real-world outcomes consistent with the same global forcing. This aim is a focus of future research.

Furthermore, we consider projections of future water consumption based only on population (stationary per capita water consumption). We choose a middle-of-the-road profile of unchanging per capita water demand, as we expect poor countries to increase their water intensities, and wealthy countries to plateau or decrease [55]. There is a rich literature on the challenge of modeling future per capita water demand [19, 55]. Given our focus here on the uncertainty arising from climate variability, future work could explore alternate per capita water demand assumptions, which would modify the risks we identify here.

Dependent on the social, political, and economic features of a basin, population change may not be the most important predictor for future basin-scale water consumption. For example, in the developing world, per capita energy and water demands are likely to increase alongside increasing living standards, suggesting that our estimate is conservative for those basins. However, complicating such an interpretation are the dynamics of how mass urbanization may shift agricultural activities (the largest source of water demand) to other basins. In contrast to the developing world, California’s per capita water consumption has decreased slightly over recent decades despite population growth due to water efficiency measures. Such unknowns would greatly influence the risks we present for some basins, as would any set of future water policies. In the present study, we aim to identify irreducible uncertainty conditional on business-as-usual, rather than introduce inscrutable uncertainties from complicated water use scenarios. Both the signal and noise associated with hydroclimate change outpace the signal and noise from population-based projections of water consumption.

The hydroclimatic response to global warming implies that the most likely global-scale outcome in the RCP8.5 forcing pathway is one of increasing population exposure to sub-annual hydroclimatic deficits requiring management responses (figures 2(e), 3, S3). Internal variability nevertheless exerts a large influence on future hydroclimate, and therefore on future population exposure (figure 3). Depending on the SSP, between 78%–80% of people are projected to live in basins where the ensemble does not robustly agree on the direction of change, and the majority of basins face possible increased or decreased net deficits as a consequence of internal climate variability interacting with anthropogenically-forced change.

Quantifying the uncertainty arising from internal variability is critical for identifying the distribution of potential real-world outcomes on which to base climate impact analyses. The model physics in the CESM influences the irreducible uncertainty in the LENS. To the extent that CESM captures real-world climate processes, our analysis of the single-model ‘irreducible uncertainty’ provides one such estimate. Ideally, there would be initial condition ensembles completed for each CMIP, and or hydrological or water resource model, as the uncertainty range within each model will be impacted by different modeling choices that create model biases in P and ET (e.g. parameterization of processes associated with clouds, convection, soil moisture, and vegetation). Furthermore, future versions of land surface components to Earth system models will likely be better positioned to simulate sub-basin and sub-annual surface hydrology with greater fidelity, allowing for large ensemble analysis of endogenous surface hydrological fields.

Despite the additional uncertainty characterizations mentioned above, the range of hydroclimatic variability identified in our results is fundamental for decision-making, as it influences the set of potential real-world outcomes around a climate trajectory, and thus the set of adaptive responses that must be considered. We have demonstrated that while projected probabilities point to increasing hydroclimatic deficits, irreducible uncertainty makes many outcomes possible under the same forcing scenario. Identifying the scope of this uncertainty is therefore a critical means for informing adaptive management practices and robust adaptation [23] in integrated water resources management [54]. The irreducible range of outcomes we have identified also has important implications for water storage in and on the land surface, as well as for social, political, cultural, and economic approaches to providing water during times of natural deficit. Recent hydroclimatic disasters suggest that existing infrastructure, governance, and management practices may not be optimally adapted to *present* climate variability, meaning that irreducible uncertainty as we have characterized in this study must be factored into climate risk management practices both in the present and the future [56].

## Supplementary Material

Supplementary Data

## Figures and Tables

**Table 1.** Global-mean basin deficits in the present and future climates. For all SSPs, we test the difference in the distribution of the 676 basins for the magnitude and duration of natural and net deficits and their differences, as well as basin-average precipitation, evapotranspiration, population, and demand, reporting the value of the global mean difference in the present and future periods (see empirical distribution functions in figure S2). We present three tests of significant differences, the two-tailed Welch’s T-test (significant difference in means at *p* < 0.05 are denoted ‘*’), the two-tailed Mann-Whitney test (significant difference in distribution centers at *p* < 0.05 are denoted ‘†’), and the bootstrapped two-tailed Kolmogorov-Smirnov test (significant difference in distributions at *p* < 0.05 are denoted ‘§’). Bolded values indicate significance in at least two of the three significance tests.

Variable	Present 1948–2010	Future 2060			Difference significance (Present v. 2060)		
**1. Natural deficit magnitude [%]**	11.2		39.9			28.7*†§	
**2. Net deficit magnitude [%]**	22.0	SSP2 67.1	SSP3 75.6	SSP5 61.8	**SSP2** 45.1*†§	**SSP3** 53.6*†§	**SSP5** 39.8*†§
**3. Difference significance, (2) v. (1) [pp]**	10.7*	SSP2 27.2	SSP3 35.7	SSP5 21.9			
**4. Natural deficit duration [mos.]**	2		2.5			0.5*†§	
**5. Net deficit duration [mos.]**	2.3	SSP2 2.8	SSP3 2.8	SSP5 2.8	**SSP2** 0.5*†§	**SSP3** 0.5*†§	**SSP5** 0.5*†§
**6. Difference significance, (5) v. (4) [mos.]**	0.3*	SSP2 0.3*	**SSP3** 0.3*§	SSP5 0.3*			
**7. Precipitation [mm/mo.]**	74.5		79.2			4.7	
**8. Evapotranspiration [mm/mo.]**	42.2		46.0			3.8*†§	
**9. Population [M]**	9.8	SSP2 13.2	SSP3 14.9	SSP5 12.0	SSP2 3.4§	**SSP3** 5.1*§	SSP5 2.2§
**10. Demand [mm/mo.]**	0.75	SSP2 1.14	SSP3 1.28	SSP5 1.05	SSP2 0.39*§	**SSP3** 0.53*§	**SSP5** 0.3*§
