# Supplementary material for: Influence of internal variability on population exposure to hydroclimatic changes
Source: Environ Res Lett. Author manuscript; Available in PMC 2020 Aug 25. (PMC7446950; doi:10.1088/1748-9326/aa5efc)
Supplement: Supplementary Data [file NIHMS960917-supplement-Supplementary_Data.pdf]

Supplemental Material for:

Influence of internal variability on population exposure to hydroclimatic changes

Justin S. Mankin[a,b,c*], Daniel Viviroli[d], Mesfin M. Mekonnen[e], Arjen Y. Hoekstra[e], Radley M. Horton[f,b], Jason E. Smerdon[a], and Noah S. Diffenbaugh[g,h]

[a] Lamont-Doherty Earth Observatory, Columbia University, New York, USA

[b] NASA Goddard Institute for Space Studies, New York, USA

[c] Emmett Interdisciplinary Program in Environment & Resources, Stanford University, California, USA

[d] Department of Geography, University of Zurich, Zurich, Switzerland

[e] Dept. of Water Engineering & Management, University of Twente, Enschede, The Netherlands

[f] Center for Climate Systems Research, Columbia University, New York, USA

[g] Department of Earth System Science, Stanford University, California, USA

[h] Woods Institute for the Environment, Stanford University, California, USA

*** Corresponding author:**
Justin S. Mankin
Ocean and Climate Physics
Lamont-Doherty Earth Observatory
Columbia University
61 Route 9W, P.O. Box 1000
Palisades, NY 10964, USA
Email: jsmankin@ldeo.columbia.edu

1 Supplemental Methods

1.1 Basin-scale analysis

We modify the STN-30p hydrological basin demarcations following the methods of ref. 1, aggregating smaller basins, particularly along coastlines, into larger, physically-meaningful basins using the coastal basins provided by ref. 2. We also mask small basins for which the GLDAS baseline data and the CESM LENS are too coarse, giving 676 distinct catchments. This is method is also consistent with ref. 3.

The GLDAS-2 baseline data provides a closure of the water budget at the basin and annual scales (Fig. S1d) consistent with theory and provides a good basis on which to estimate historical deficits.

1.2 Projections of net deficits

We assess the CESM LENS monthly and annual basin-scale trends in P and ET against the GLDAS-2 baseline for 1948-2010 (Fig. S1a). We highlight the basins bounded by the range of the ensemble in dark grey, and those not bounded by the ensemble with the absolute magnitude of their bias (mm/day) (Fig. S1b,c). The CESM LENS performs well at characterizing basin-scale hydroclimatic trends over the 20th century, denoted by the small difference in baseline trends, giving us confidence to use CESM basin-scale trend projections to estimate future hydroclimate. Each LENS ensemble member represents a combination of forced change and unforced variability. We estimate monthly-scale trends in P and ET from the CESM LENS from 2011-2080, expressing them as relative changes (% change per 50-years, thus change at ~2060). The differences in trends among the ensemble arise from persistent atmospheric noise.

Following ref. 3, we correct for any minor systematic biases in the LENS baseline simulation (Fig. S1): we first multiply relative monthly trends (%) for each ensemble member by the GLDAS-2 baseline monthly mean value for P and ET (mm). These absolute monthly mean changes are then added to the GLDAS-2 baseline monthly means, providing estimates of future P and ET for each month in each basin for each ensemble member. We then calculate the net deficits (as described in the manuscript *Methods* section), and difference the future net deficits from the observed net deficits. To highlight the importance of climate variability in driving the range of uncertainty we show the spatial pattern of future change in natural hydroclimatic deficits (not considering any scenario of human water demand). We show the full range of the 30-member ensemble, highlighting the ensemble minimum, mean and maximum changes across the ensemble for each basin (Fig. S3) as well as the ensemble minimum and maximum combination of P and ET, to include their statistical significances (Fig. S5).

1.3 Statistical tests

We test the global difference in natural and net deficits using three statistical tests (one parametric and two nonparametric): (1) a two-tailed Welch's t-test of equal means (used when the data samples have unequal variances); (2) a two-tailed Mann-Whitney-Wilcoxon test of equal distribution centers (under the assumption that data are not Gaussian); and (3) a bootstrapped two-tailed Kolmogorov-Smirnov test of equal probability distributions. We reject the null hypotheses of equal values using $\alpha=0.05$. Results from these tests are presented in Table 1. We bold those quantities that are significant in two of the three tests.

We perform several different statistical tests at the basin-scale, depending on the quantity presented. All linear time trends have autocorrelation-corrected two-tailed Student's test rejecting p>0.05, following ref. [4] (Fig. S5). Significance for ensemble-mean trends is presented as the ensemble signal-to-noise (Fig. 2a,b), where significant change is an ensemble-mean response greater than ensemble variability. To test for significant ensemble-mean change in basin net deficits, we employ a one-tailed Student's t-test, rejecting the null hypothesis if the ensemble-mean change (relative to the GLDAS-2, presented in Fig. 1) is insignificant at the 5% level (Fig. 2e, S3). The basin-scale risks presented in Figs. 2e and S3 serve as a final robustness test. We present the percent of the ensemble agreeing on the sign of change. We present the population exposed to varied levels of risk in net deficit magnitudes and durations in Tables S1 and S2.

2 Supplemental Results and Discussion

2.1 The importance of the LENS characterization of uncertainty

The CESM LENS ensemble allows analyses that seek to identify the range of uncertainty from internal climate variability. This is in contrast to multi-model projections that allow estimates of response uncertainty (namely, how model physics influence model response). In multi-model ensembles, such as the CMIP5 there are not enough simulations with each individual model to effectively sample each model's representation of internal variability. This can be seen in Fig. S5: the CESM can have statistically-significant trends of opposite signs at basin-scales arising from internal variability in the same forcing pathway and thus a confounding picture of the response from forcing.

Impacts analyses have largely focused on “best-estimate predictions” to aid adaptation decision-making. The requirement of robust decision-making under uncertainty[8] nevertheless requires the identification of adaptation strategies that produce benefits under the broadest range of climate outcomes[9], such as those from internal variability. “Uncertainty means more than one outcome is consistent with expectations”[10], and expectations are shaped by what we identify to be the full range of possible outcomes from internal variability (not those outcomes arising from differing parameterization choices in multi-model ensembles). This suggests that poorly-characterized estimates of internal variability from multi-model ensembles could bias expectations of global warming.

Adaptations to hydroclimate changes will likely require regional and local responses encompassing institutional, social, and economic changes[4], and therefore require significant time horizons for effective planning. Because adaptations are best made proactively to offset damages, however, their implementation should necessary occur when internal variability has the potential to mask or reverse long-term trends. This can create the appearance of maladaptation or be truly maladaptive and thus increase vulnerability to climate extremes. Furthermore, such adaptations will need benchmarks for evaluation as part of a strategy of robust decision-making under uncertainty. Many of these benchmarks are a function of the climate system itself, such as when impacts manifest, which are subject to irreducible uncertainty from internal variability.

2.2 Net deficits

Our quantification of future net deficits is a simple and transparent method to estimate future change in water availability with large ensembles of climate models and scenarios of population growth. To avoid over-speculation of future pathways in water consumption, we assume that per capita water consumption at the basin-scale remains stationary; that is, population projections are the only driver of water consumption changes. While this increase in population may come with improved water-use efficiency, increasing population combined with changes in diet and water use preferences in the developing world could increase per capita consumption that would in turn increase global-scale net deficits within a given climate pathway[11]. Second, because our measure of net deficits is based on long-run sub-annual climatological relationships between water supply (P) and water demand (ET-H), it highlights climatological changes to sub-annual variability, and thus deficits. Because variability on shorter or longer timescales can also play an important role in local water stress, the long-run mean projections that we show can potentially encompass large shifts in short-term hydroclimate variability and therefore management responses.

2.3 Projected trends in hydroclimate

The spatial pattern of the CESM LENS ensemble-mean annual basin-scale precipitation response (Fig. 2a) is similar to the CMIP5 ensemble response in the mid-21st-century of the RCP8.5 pathway (Figure 3, Box CC-RC from ref. [7]; Figure AI.SM8.5.9 from ref. [12]). The CESM LENS shows consistent 50-year decreases in precipitation along southwestern Australia (~8-12%), South Africa (12-18%), western Patagonia (12-14%), northeastern Brazil and the Amazon basin (2-18%), western Sahara

(2-10%) and the Mediterranean (2-4%), as well as robust mid- and high-latitude increases over Eurasia (Fig. 2a,c). The magnitude and robustness of the decreases are heavily influenced by the LENS expression of internal variability (Fig. 2c): in many of these regions responses are not robust across the ensemble (S/N<1), denoted by the stippling in those basins (Fig. 2a).

Annual evapotranspiration projections show a similar spatial pattern to both the CESM change in precipitation (Fig. 2a) and the late-21st-century CMIP5 ensemble change in evaporation under the RCP8.5 (Figure 12.25 from ref. 6). For example, there are marked, but not robust (S/N<1), warming-induced ET increases over eastern and southeastern Europe by 2060. These eastern and southeastern European increases in ET are collocated with small or negative precipitation trends, which are consistent with increases in the extent of the Hadley Circulation[6,13,14].

We present a scatterplot of the maximum and minimum single-realization combination of P and ET change, as well as the ensemble-mean response, and their associated natural deficits (Fig. S5). Multi-decadal hydroclimatic variability creates the potential for statistically significant trends that deviate far from the ensemble mean response. Increases in annual-scale ET that exceed increases in annual-scale P drive increases in net deficits (Fig. S5a), as do decreases in annual-scale P or ET (Fig. S5a. c). The annual-scale changes in P and ET generally indicate a basin's drying or wetting (i.e., its change in net deficits that are estimated based on monthly changes), as seen by the colors preferentially falling on one side of the line of no change (1:1 line, Fig. S5b), consistent with expectation[15]. The consistent relationship between annual-scale P and ET trends and sub-annually-calculated changes in net deficits, allows us to present the

annual-scale trends as a proxy for the direction of net deficit change (as presented in Fig. 2 and in the main).

References

1. Viviroli, D., Dürr, H. H., Messerli, B., Meybeck, M. & Weingartner, R. Mountains of the world, water towers for humanity: Typology, mapping, and global significance. *Water Resour. Res.* **43,** W07447 (2007).
2. Meybeck, M., Dürr, H. H. & Vörösmarty, C. J. Global coastal segmentation and its river catchment contributors: A new look at land-ocean linkage. *Global Biogeochem. Cycles* **20,** 1–15 (2006).
3. Mankin, J. S., Viviroli, D., Singh, D., Hoekstra, A. Y. & Diffenbaugh, N. S. The potential for snow to supply human water demand in the present and future. *Environ. Res. Lett.* **10,** 114016 (2015).
4. Mankin, J. S. & Diffenbaugh, N. S. Influence of temperature and precipitation variability on near-term snow trends. *Clim. Dyn.* **45,** 1099–1116 (2014).
5. Paté-Cornell, M. E. Uncertainties in risk analysis: Six levels of treatment. *Reliab. Eng. Syst. Saf.* **54,** 95–111 (1996).
6. Collins, M. *et al.* Long-term Climate Change: Projections, Commitments and Irreversibility. *Clim. Chang. 2013 Phys. Sci. Basis. Contrib. Work. Gr. I to Fifth Assess. Rep. Intergov. Panel Clim. Chang.* (2014).
7. Diffenbaugh, N. S. *et al.* in *Climate Change 2014: Impacts, Adaptation, and Vulnerability. Part A: Global and Sectoral Aspects. Contribution of Working Group II to the Fifth Assessment Report of the Intergovernmental Panel on Climate Change* (eds. Field, C. B. et al.) 137–141 (Cambridge University Press, 2014).
8. Lempert, R. J. & Collins, M. T. Managing the risk of uncertain threshold responses: comparison of robust, optimum, and precautionary approaches. *Risk Anal. an Off. Publ. Soc. Risk Anal.* **27,** 1009–26 (2007).
9. Lempert, R. J. Scenarios that illuminate vulnerabilities and robust responses. *Clim. Change* **117,** 627–646 (2012).
10. Dessai, S., Hulme, M., Lempert, R. J. & Pielke Jr, R. A. in *Adapting to climate change: thresholds, values, governance* (eds. Adger, N., Lorenzoni, I. & O'Brien, K.) 64–78 (Cambridge University Press, 2009).
11. Ercin, A. E. & Hoekstra, A. Y. Water footprint scenarios for 2050: A global analysis. *Environ. Int.* **64,** 71–82 (2014).
12. van Oldenborgh, G. J. *et al.* Annex I: Atlas of Global and Regional Climate Projections. *Clim. Chang. 2013 Phys. Sci. Basis. Contrib. Work. Gr. I to Fifth Assess. Rep. Intergov. Panel Clim. Chang.* 1–159 (2013).
13. Lau, W. K. M. & Kim, K.-M. Robust Hadley Circulation changes and increasing global dryness due to CO2 warming from CMIP5 model projections. *Proc. Natl. Acad. Sci.* 201418682 (2015). doi:10.1073/pnas.1418682112
14. Sherwood, S. & Fu, Q. A Drier Future? *Science* **343,** 737–739 (2014).
15. Greve, P. *et al.* Global assessment of trends in wetting and drying over land. *Nat.*

Geosci. **7,** (2014).

3 Supplemental figure captions

Fig. S1 Data validation. **a,** Comparison of distributions of historical (1948-2010) monthly precipitation (P, blue) and evapotranspiration (ET, red) trends in the GLDAS data (solid lines) and the CESM LENS (dotted lines). **b-c,** Historical annual trend difference maps for precipitation [b] and evapotranspiration [c]. In [b] and [c] for the basins with GLDAS trends not bounded by the LENS trends, we show the magnitude of the difference between the GLDAS trend and the maximum or minimum LENS trend. Dark grey basins have GLDAS trends that fall within the distribution of LENS trends. **d,** The annual-scale, basin-scale water balance (P-ET-Q≈0) in the GLDAS data for 1948-2010.

Fig. S2 Global deficits in the present and future climates. **a,** Empirical cumulative distribution functions for the magnitude of natural deficits and net deficits (mm) in the present and future climates. We show the ensemble mean ECDF for SSP5, though the other SSP distributions stack atop the P-ET-H future distributions. **b,** As in [a], but for the duration of deficits (months).

Fig. S3 As in Fig. 2e, the risk of net deficit increases for all three SSP scenario in magnitude ([a]-[c], left column) and duration ([d]-[f], right column). The magnitudes are calculated as a percent of annual precipitation. We apply a threshold for inclusion for each run in each basin: the absolute magnitude of net deficit change must be greater than present-day water storage infrastructure in the GRanD database. Hatched basins have insignificant ensemble mean changes ($p>0.05$) based on a one-sample Student's t-test.

Fig. S4 Full range of projected changes in magnitude [a-d] and duration [d-f] of natural deficits (P-ET<0), not including human demand. These figures reflect forced climate and climate variability only. **a,e,** Ensemble composite of the basin-maximum change in magnitude (mm) and duration (months) of net deficit projected for each basin as a difference from the 1948-2010 baseline. **b,f,** As in [a,d], but for the basin-mean change in net deficit. Stippling in [b,f] indicates insignificant mean change (p>0.05) based on a single-sample Student's t-test. **c,g,** As in [a,d], but for the basin-minimum change. **d,h,** The difference between [a,c] and [e,g], showing the full range of possible change within the LENS. Note that [a,e] and [e,g] are composites from the ensemble and not a single realization.

Fig. S5 Basin scale internal variability in P and ET trends. We show annual mean trends in precipitation and evapotranspiration estimated from 2011-2080 from the CESM LENS, for: **a,** each basin's maximum single-run combination of 50-year precipitation and evapotranspiration trends, **b,** each basin's ensemble mean combination, and **c,** each basin's single-run minimum combination. Colors are the projected change in the magnitudes of natural deficit for that P and ET combination (ensemble member) and basin (% of annual precipitation). Large open circles in [a] and [c] indicate basins where both the P and ET trends are statistically significant (p<0.05), whereas the large open circles in [b] are where the ensemble mean exceeds one standard deviation of the ensemble variability. Small circles are for trends that are not significant. We annotate [b]

with the classification of Greve et al. 2014, highlighting that general relationship of values falling on either side of the 1:1 line (no change), show expected changes in integrated deficits and thus are physically meaningful measures of wetting and drying. Natural (and net) deficits are calculated off of monthly trends (rather than the annual trends presented here), highlighting that annual-scale changes in P and ET can inform the direction of change in deficits. Finally, statistically-significant trends far from the ensemble mean is possible, denoted by the many large circles in [a] and [c].

Table captions

Table S1 Probability (as percent of simulations, with corresponding population, and basins exposed) of any increase in net deficit magnitude greater than present-day water storage infrastructure in the GRanD database.

Table S2 Probability (as percent of simulations, with corresponding population percentages, and basins exposed) of any increase in net deficit durations, but whose magnitude is greater than present-day water storage infrastructure in the GRanD database.

PROBABILITY	SSP2 population (# of basins)	SSP3 population (# of basins)	SSP5 population (# of basins)
**>3%**	87 (266)	87 (268)	87 (266)
**45-55%**	12 (45)	22 (43)	7 (38)
**>66%**	21 (78)	20 (82)	22 (81)
**<66%**	79 (598)	80 (594)	78 (595)
**>75%**	17 (56)	17 (55)	17 (54)
**<25%**	32 (462)	33 (462)	44 (467)
**>50%**	31 (125)	35 (131)	31 (122)
**<50%**	66 (535)	61 (529)	68 (541)
**>90%**	10 (16)	10 (18)	7 (18)
**100%**	<1 (2)	5 (3)	1 (3)

Table S1 Probability (as percent of simulations, with corresponding population, and basins exposed) of any increase in net deficit magnitude greater than present-day water storage infrastructure in the GRanD database.

PROBABILITY	SSP2 % of global population (# of basins)	SSP3 % of global population (# of basins)	SSP5 % of global population (# of basins)
**>3%**	83 (265)	84 (267)	84 (265)
**45-55%**	10 (41)	13 (39)	9 (33)
**>66%**	20 (70)	21 (71)	19 (33)
**<66%**	79 (606)	78 (605)	79 (610)
**>75%**	13 (44)	17 (45)	16 (47)
**<25%**	45 (468)	32 (466)	44 (469)
**>50%**	29 (118)	31 (121)	28 (112)
**<50%**	68 (550)	62 (543)	69 (554)
**>90%**	9 (13)	8 (12)	5 (13)
**100%**	<1 (1)	5 (2)	1 (2)

Table S2 Probability (as percent of simulations, with corresponding population percentages, and basins exposed) of any increase in net deficit durations, but whose magnitude is greater than present-day water storage infrastructure in the GRanD database.

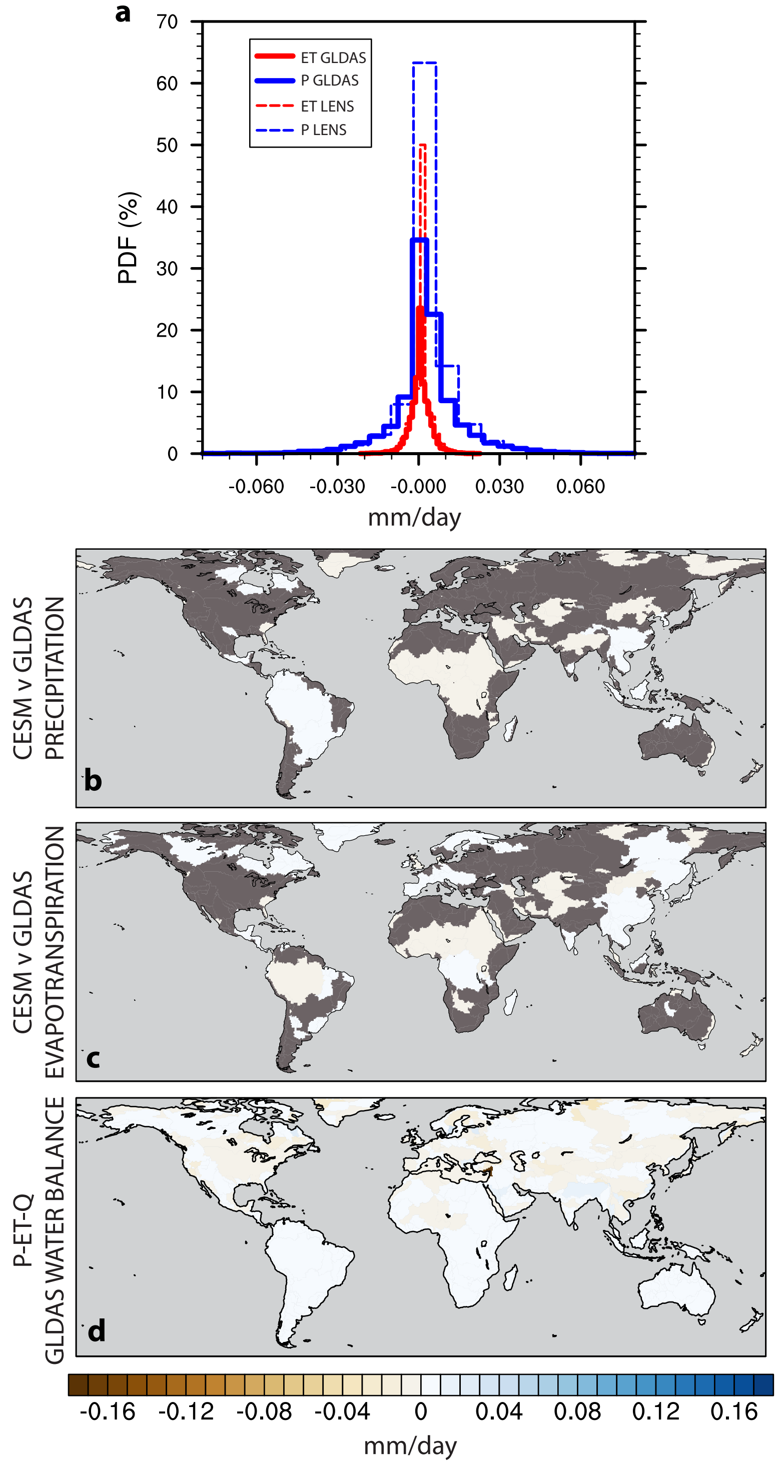
a
70
60
50
40
30
20
10
0
PDF (%)
ET GLDAS
P GLDAS
ET LENS
P LENS
-0.060
-0.030
-0.000
0.030
0.060
mm/day
CESM v GLDAS
PRECIPITATION
b
CESM v GLDAS
EVAPOTRANSPIRATION
c
P-ET-Q
GLDAS WATER BALANCE
d
-0.16
-0.12
-0.08
-0.04
0
0.04
0.08
0.12
0.16
mm/day

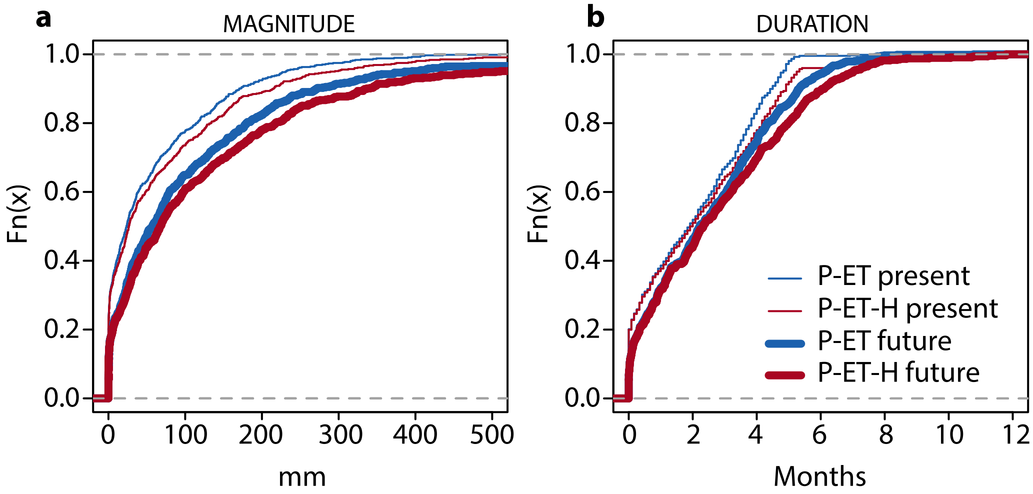

a
MAGNITUDE
1.0
0.8
0.6
0.4
0.2
0.0
Fn(x)
0
100
200
300
400
500
mm
b
DURATION
1.0
0.8
0.6
0.4
0.2
0.0
Fn(x)
0
2
4
6
8
10
12
Months
P-ET present
P-ET-H present
P-ET future
P-ET-H future

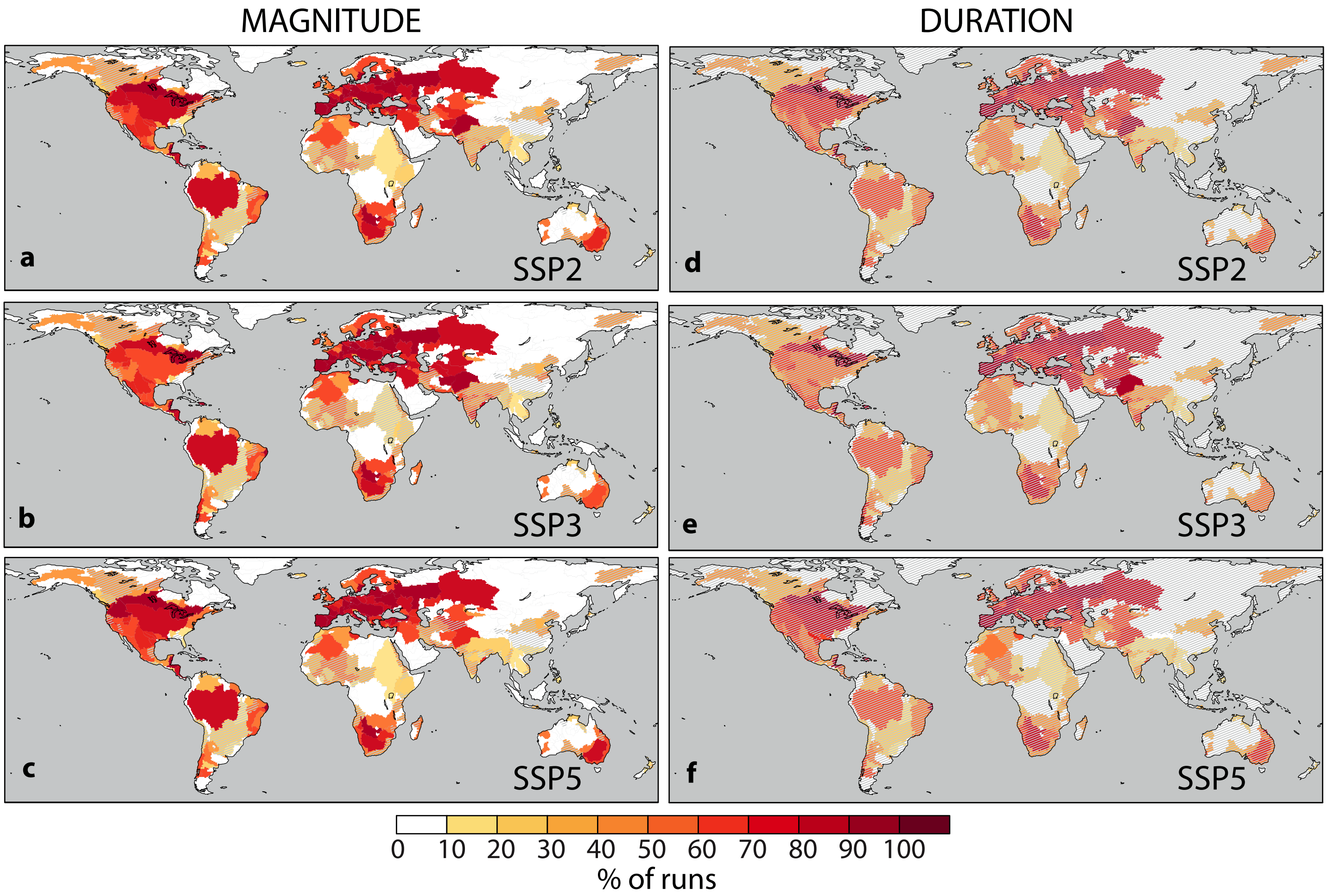
MAGNITUDE
DURATION
a
SSP2
d
SSP2
b
SSP3
e
SSP3
c
SSP5
f
SSP5
0 10 20 30 40 50 60 70 80 90 100
% of runs

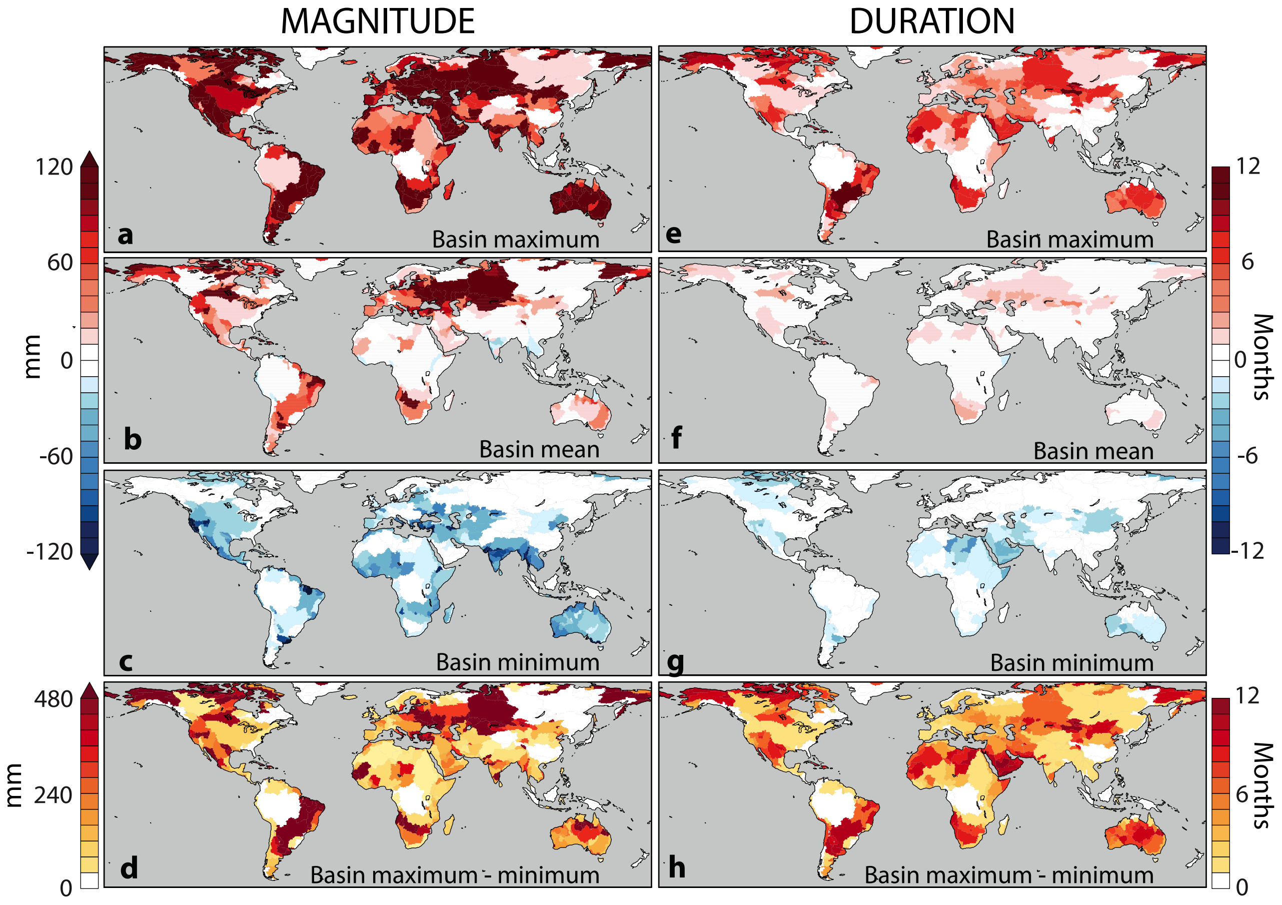

MAGNITUDE
DURATION
a
Basin maximum
b
Basin mean
c
Basin minimum
d
Basin maximum - minimum
e
Basin maximum
f
Basin mean
g
Basin minimum
h
Basin maximum - minimum
120
60
0
-60
-120
mm
480
240
0
mm
12
6
0
-6
-12
Months
12
6
0
Months

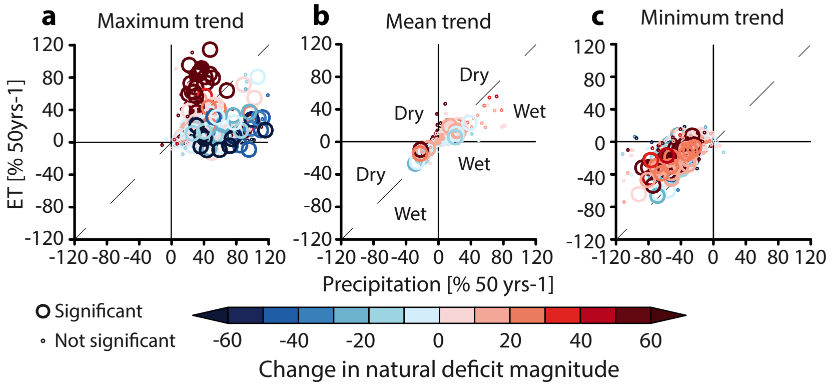
a
Maximum trend
b
Mean trend
c
Minimum trend
ET [% 50yrs-1]
Precipitation [% 50 yrs-1]
Dry
Wet
120
80
40
0
-40
-80
-120
Significant
Not significant
-60
-40
-20
0
20
40
60
Change in natural deficit magnitude
